# Supplementary material for: Modelling Skylarks (Alauda arvensis) to Predict Impacts of Changes in Land Management and Policy: Development and Testing of an Agent-Based Model
Source: PLoS One. 2013 Jun 6;8(6):e65803. doi: 10.1371/journal.pone.0065803 (PMC3675089; doi:10.1371/journal.pone.0065803)
Supplement: Supporting Information S4 — The skylark ODdox as a zipped archive. (ZIP) [file pone.0065803.s004.zip › Skylark_ODdox/class_rodenticide_manager.html]

ALMaSS Skylark ODdox: RodenticideManager Class Reference


ALMaSS Skylark ODdox
 2.0


- Main Page
- Related Pages
- Classes
- Files

- Class List
- Class Index
- Class Hierarchy
- Class Members

Public Member Functions |
Protected Attributes

RodenticideManager Class Reference

Class for management of bait locations.
More...

`#include <Rodenticide.h>`

List of all members.

|  |  |
| --- | --- |
| Public Member Functions | |
| void | Diffuse (int a\_x, int a\_y) |
|  | Move poisoned mice around to twin map. |
| void | DistributeRing (int a\_x, int a\_y, double a\_amount) |
|  | Distribute an amount to the twin map in a ring. |
| void | DoDeath (void) |
|  | Reduce each cell in twin map by m\_deathRate. |
| void | DoDiffuse (void) |
| void | DoImmigration (void) |
| void | DoPlaceBait () |
|  | Initiates bait placement for those bait locations necessary. |
| void | DumpRodenticideMap (string a\_fname) |
|  | Dumps a map of rodenticide loads per 1m2 cells. |
| int | GetBaitStartDate (TTypesBaitLocation) |
|  | Determines when the bait will be placed within a season. |
| double | GetMap (int a\_x, int a\_y) |
|  | Get poisoned mice at x,y. |
| double | GetMapTwin (int a\_x, int a\_y) |
|  | Get poisoned mice at x,y on twin map. |
| double | GetRodenticide (unsigned a\_x, int a\_y) |
|  | Return the poisoned mice value at x,y. |
| void | InitiateRodenticidePolyData (string a\_fname) |
|  | Opens and initiates the output file for polygon rodenticide information. |
| void | RecordRodenticidePolyData (string a\_fname) |
|  | Records rodenticide loads per polygon. |
|  | RodenticideManager (string fname, Landscape \*a\_land) |
|  | RodenticideManager constructor. |
| void | SetMap (int a\_x, int a\_y, double a\_p) |
|  | Set poisoned mice at x,y. |
| void | SetMapTwin (int a\_x, int a\_y, double a\_p) |
|  | Set poisoned mice at x,y on twin map. |
| void | SetMapTwinMult (int a\_x, int a\_y, double a\_p) |
|  | Multiply poisoned mice at x,y on twin map by constant. |
| void | SetMapTwinP (int a\_x, int a\_y, double a\_p) |
|  | Add to poisoned mice at x,y on twin map. |
| bool | ShouldPlaceBait (TTypesBaitLocation) |
|  | Place bait dependent upon a frequency test. |
| void | Tick (void) |
|  | Advance one day. |
| virtual | ~RodenticideManager (void) |
|  | RodenticideManager destructor. |

|  |  |
| --- | --- |
| Protected Attributes | |
| vector< BaitLocation > | m\_BaitList |
|  | List of bait locations. |
| double | m\_deathRate |
|  | poisoned mice death rate |
| double | m\_diffusionRate |
|  | poisoned mice diffusion rate |
| int | m\_gridsize |
|  | rodenticide grid size - also affect diffusion rate |
| int | m\_height |
| int | m\_heightm1 |
| double | m\_immigrationRate |
|  | poisoned mice immigration rate |
| Landscape \* | m\_land |
|  | Landscape pointer. |
| vector< double > | m\_pmouse\_map\_main |
|  | Map of probability of encountering a poisoned mouse. |
| vector< double > | m\_pmouse\_map\_twin |
|  | Temporary map for calculation poisoned mouse diffusion. |
| int | m\_seasonstartprob [tbl\_foobar][1000] |
|  | season start probability distribution |
| int | m\_width |
|  | Landscape dimensions. |

---

## Detailed Description

Class for management of bait locations.

---

## Constructor & Destructor Documentation

|  |  |  |  |
| --- | --- | --- | --- |
| RodenticideManager::RodenticideManager | ( | string | *fname*, |
|  |  | Landscape \* | *a\_land* |
|  | ) |  |  |

RodenticideManager constructor.

Uses the file name to open a file and read in all bait locations required for managemnet

```
      Creates the rodenticide grid with its twin-map and fills them with zeros.  The maps have a resolution which is defined by __RODENTICIDE_GRID_RES. 
      Cannot have diffusion of >= map width or height - this needs checking to prevent wrapping around the world.
```

References cfg\_rodenticide\_deathRate, cfg\_rodenticide\_diffusionRate, cfg\_rodenticide\_gridsize, cfg\_rodenticide\_immigrationRate, BaitLocation::SetBLtype(), BaitLocation::SetX(), BaitLocation::SetY(), Landscape::SupplySimAreaWidth(), tbl\_foobar, CfgInt::value(), and CfgFloat::value().

{

// Read in input parameters

m\_gridsize = cfg\_rodenticide\_gridsize.value();

m\_deathRate = 1.0-cfg\_rodenticide\_deathRate.value();

m\_diffusionRate = cfg\_rodenticide\_diffusionRate.value();

m\_immigrationRate = cfg\_rodenticide\_immigrationRate.value();

ifstream ifile(a\_fname, ios::in);

if ( !ifile.is\_open() )

{

cout << "Cannot open input file " << a\_fname << endl;

char ch;

cin >> ch;

exit(1);

}

int noBaits, x, y, blt;

TTypesBaitLocation BL\_type = tbl\_foobar;

BaitLocation Bait(0,0,BL\_type);

ifile >> noBaits;

for (int i = 0; i < noBaits; i++)

{

ifile >> x >> y >> blt;

Bait.SetX(x >> m\_gridsize);

Bait.SetY(y >> m\_gridsize);

Bait.SetBLtype((TTypesBaitLocation)blt);

m\_BaitList.push\_back(Bait);

}

// Need to put actual baits out in the landscape

m\_land = a\_land;

m\_width = m\_land->SupplySimAreaWidth() >> m\_gridsize;

m\_height = m\_land->SupplySimAreaHeight() >> m\_gridsize;

m\_heightm1 = m\_height-1; // Saves many many calculations later.

m\_pmouse\_map\_main.resize((m\_width+1) \* (m\_height+1));

m\_pmouse\_map\_twin.resize((m\_width+1) \* (m\_height+1));

for (int i=0; i<m\_width+1; ++i) for (int j=0; j<m\_height+1; ++j)

{

m\_pmouse\_map\_main[j\*m\_height + i] = 0;

m\_pmouse\_map\_twin[j\*m\_height + i] = 0;

}

// read in and handle the probability distributions for seasonal start dates for bait locations

ifstream i2file("BaitLocationsSeasonalStartProbs.txt", ios::in);

if ( !ifile.is\_open() )

{

cout << "Cannot open input file " << "BaitLocationsSeasonalStartProbs.txt" << endl;

char ch;

cin >> ch;

exit(1);

}

double temp[tbl\_foobar][365];

for (int i = 0; i < 365; i++)

{

for (int j=0; j<tbl\_foobar; j++)

{

i2file >> temp[j][i];

}

}

// data exists in temp. next for each BL type fill in the probability array in fractions of 0.1%

// each entry corresponds to one day when bait might be started

for (int j=0; j<tbl\_foobar; j++)

{

int index = 0;

for (int i = 0; i < 365; i++)

{

int prob = (int) floor((temp[j][i]\*10)+0.5) + index;

for (index; index<prob; index++) m\_seasonstartprob[j][index] = i;

if (index>=1000) i=365;

}

}

InitiateRodenticidePolyData("RodenticidePolyData.txt");

}

|  |  |  |  |  |  |
| --- | --- | --- | --- | --- | --- |
| RodenticideManager::~RodenticideManager | ( | void |  | ) |  |

virtual

RodenticideManager destructor.

{

;

}

---

## Member Function Documentation

|  |  |  |  |
| --- | --- | --- | --- |
| void RodenticideManager::Diffuse | ( | int | *a\_x*, |
|  |  | int | *a\_y* |
|  | ) |  |  |

inline

Move poisoned mice around to twin map.

{

double before = GetMap(a\_x, a\_y);

if (before < 0.0001)

{

SetMapTwinP(a\_x, a\_y,0); // Prevents infinitely small calculations

return;

}

double after = before \* m\_diffusionRate;

double diff\_out = after/8;

SetMapTwinP(a\_x, a\_y,before-after);

DistributeRing(a\_x,a\_y,diff\_out);

}

|  |  |  |  |
| --- | --- | --- | --- |
| void RodenticideManager::DistributeRing | ( | int | *a\_x*, |
|  |  | int | *a\_y*, |
|  |  | double | *a\_amount* |
|  | ) |  |  |

inline

Distribute an amount to the twin map in a ring.

Distribute a\_amount to the eight cells around m\_x,m\_y

{

int x1 = a\_x - 1;

int y1 = a\_y - 1;

int y2 = y1 + 2;

int x2 = x1 + 2;

if ((y1<0) || (y2==m\_height) || (x1<0) || (x2==m\_width))

{

// We need to do this to prevent the diffusion building a wall around the edge, but it could be assessed as to whether the speed penalty is worth it

x1 = (x1 + m\_width) % m\_width;

x2 = (x2 + m\_width) % m\_width;

y1 = (y1 + m\_height) % m\_height;

y2 = (y2 + m\_height) % m\_height;

}

SetMapTwinP(x1,y1,a\_amount);

SetMapTwinP(x1,y2,a\_amount);

SetMapTwinP(x1,a\_y,a\_amount);

SetMapTwinP(x2,y1,a\_amount);

SetMapTwinP(x2,y2,a\_amount);

SetMapTwinP(x2,a\_y,a\_amount);

SetMapTwinP(a\_x,y1,a\_amount);

SetMapTwinP(a\_x,y2,a\_amount);

}

|  |  |  |  |  |  |
| --- | --- | --- | --- | --- | --- |
| void RodenticideManager::DoDeath | ( | void |  | ) |  |

inline

Reduce each cell in twin map by m\_deathRate.

{

for (int i=0;i<m\_width; i++)

for (int j=0; j<m\_height; j++) SetMapTwinMult(i,j,m\_deathRate);

}

|  |  |  |  |  |  |
| --- | --- | --- | --- | --- | --- |
| void RodenticideManager::DoDiffuse | ( | void |  | ) |  |

inline

{

for (int i=0;i<m\_width; i++)

for (int j=0; j<m\_height; j++) Diffuse(i,j);

}

|  |  |  |  |  |  |
| --- | --- | --- | --- | --- | --- |
| void RodenticideManager::DoImmigration | ( | void |  | ) |  |

{

unsigned sz = (unsigned) m\_BaitList.size();

for (unsigned i=0; i<sz; i++)

{

if (m\_BaitList[i].GetMass()>0)

{

m\_BaitList[i].ReduceMass(1); // subtract 1 from mass

int x = m\_BaitList[i].GetX();

int y = m\_BaitList[i].GetY();

m\_pmouse\_map\_main[y\*m\_width + x] += m\_immigrationRate;

}

}

}

|  |  |  |  |  |  |
| --- | --- | --- | --- | --- | --- |
| void RodenticideManager::DoPlaceBait | ( | void |  | ) |  |

Initiates bait placement for those bait locations necessary.

Depending upon the bait type, whether the bait has been used this year and whether it is destined to be used this method will add mass to the bait locations.   
Housekeeping on Jan 1st is to remove all masses and zero all flags for the start of the new year.   
The first test is of date, since whether a BL is used is determined by the frequency of that BL type on Jan 1st.   
The next test is on the season, determining the start of bait placement.   
NB this not so time critical so no optimising of the loops is done here.

References cfg\_rodenticide\_BLcountry\_Length, cfg\_rodenticide\_BLtown\_Length, cfg\_rodenticide\_BLwoodland\_Length, g\_msg, tbl\_country, tbl\_town, tbl\_woodland, CfgInt::value(), MapErrorMsg::Warn(), and WARN\_FILE.

{

int day = m\_land->SupplyDayInYear();

unsigned sz = (unsigned) m\_BaitList.size();

if (day==0)

{

for (unsigned i=0; i<sz; i++)

{

m\_BaitList[i].Reset();

if (ShouldPlaceBait(m\_BaitList[i].GetBLtype()))

{

m\_BaitList[i].SetUseFlag(true);

m\_BaitList[i].SetstartDay(GetBaitStartDate(m\_BaitList[i].GetBLtype()));

}

}

}

// Loop through all the BLs and initiate any needed doing today

for (unsigned i=0; i<sz; i++)

{

if (m\_BaitList[i].GetUseFlag())

{

if (m\_BaitList[i].GetstartDay() == day )

{

// Due to start today, so set bait mass & unset use flag because we have used it now.

// Find the bait mass

int mass;

switch (m\_BaitList[i].GetBLtype())

{

case tbl\_town:

mass = cfg\_rodenticide\_BLtown\_Length.value();

break;

case tbl\_country:

mass = cfg\_rodenticide\_BLcountry\_Length.value();

break;

case tbl\_woodland:

mass = cfg\_rodenticide\_BLwoodland\_Length.value();

break;

default:

g\_msg->Warn( WARN\_FILE, "RodenticideManager::DoPlaceBait(void): Unknown bait type","");

exit( 0 );

}

m\_BaitList[i].SetMass(mass);

m\_BaitList[i].SetUseFlag(false);

// Add the bait to the main map

SetMap(m\_BaitList[i].GetX(),m\_BaitList[i].GetY(),1.0);

}

}

}

}

|  |  |  |  |  |  |
| --- | --- | --- | --- | --- | --- |
| void RodenticideManager::DumpRodenticideMap | ( | string | *a\_fname* | ) |  |

Dumps a map of rodenticide loads per 1m2 cells.

Uses the file name to open a file and saves the rodenticide value for each cell at the time this method is called.

{

ofstream ifile(a\_fname, ios::out);

if ( !ifile.is\_open() )

{

cout << "Cannot open output file for rodenticide map dump " << a\_fname << endl;

char ch;

cin >> ch;

exit(1);

}

int xext = m\_land->SupplySimAreaWidth();

int yext = m\_land->SupplySimAreaHeight();

ifile << "Width: "<< xext << endl;

ifile << "Height: "<< yext << endl;

for (int y=0; y<yext; y++)

{

for (int x=0; x<xext; x++)

{

ifile << m\_land->SupplyRodenticide(x,y) << '\t';

}

ifile << endl;

}

ifile.close();

}

|  |  |  |  |  |  |
| --- | --- | --- | --- | --- | --- |
| int RodenticideManager::GetBaitStartDate | ( | TTypesBaitLocation | *a\_BLt* | ) |  |

Determines when the bait will be placed within a season.

References g\_rand\_uni.

{

int chance = (int) floor((g\_rand\_uni() \* 1000)+0.5);

return m\_seasonstartprob[a\_BLt][chance];

}

|  |  |  |  |
| --- | --- | --- | --- |
| double RodenticideManager::GetMap | ( | int | *a\_x*, |
|  |  | int | *a\_y* |
|  | ) |  |  |

inline

Get poisoned mice at x,y.

{ return m\_pmouse\_map\_main[a\_y\*m\_width + a\_x]; }

|  |  |  |  |
| --- | --- | --- | --- |
| double RodenticideManager::GetMapTwin | ( | int | *a\_x*, |
|  |  | int | *a\_y* |
|  | ) |  |  |

inline

Get poisoned mice at x,y on twin map.

{ return m\_pmouse\_map\_twin[a\_y\*m\_width + a\_x]; }

|  |  |  |  |
| --- | --- | --- | --- |
| double RodenticideManager::GetRodenticide | ( | unsigned | *a\_x*, |
|  |  | int | *a\_y* |
|  | ) |  |  |

inline

Return the poisoned mice value at x,y.

Referenced by Landscape::SupplyRodenticide().

{ return m\_pmouse\_map\_main[(a\_x >> m\_gridsize) + (a\_y >> m\_gridsize)\*m\_width]; }

|  |  |  |  |  |  |
| --- | --- | --- | --- | --- | --- |
| void RodenticideManager::InitiateRodenticidePolyData | ( | string | *a\_fname* | ) |  |

Opens and initiates the output file for polygon rodenticide information.

Uses the file name to open a file and saves the rodenticide value for each polygon at the time this method is called.

{

ofstream ofile(a\_fname, ios::out);

if ( !ofile.is\_open() )

{

cout << "Cannot open output file for rodenticide poly initiate " << a\_fname << endl;

char ch;

cin >> ch;

exit(1);

}

int noPoly=m\_land->SupplyNumberOfPolygons();

ofile << noPoly << endl;

for (int p=0; p<(noPoly-1); p++)

{

ofile << m\_land->SupplyPolyRefVector(p) << '\t';

}

ofile << m\_land->SupplyPolyRefVector(noPoly-1) << endl;

for (int p=0; p<(noPoly-1); p++)

{

ofile << m\_land->SupplyElementTypeFromVector(p) << '\t';

}

ofile << m\_land->SupplyElementTypeFromVector(noPoly-1) << endl;

for (int p=0; p<(noPoly-1); p++)

{

int area = m\_land->SupplyPolygonAreaVector(p);

ofile << area << '\t';

}

ofile << m\_land->SupplyPolygonAreaVector(noPoly-1);

ofile.close();

}

|  |  |  |  |  |  |
| --- | --- | --- | --- | --- | --- |
| void RodenticideManager::RecordRodenticidePolyData | ( | string | *a\_fname* | ) |  |

Records rodenticide loads per polygon.

Uses the file name to open a file and saves the rodenticide value for each polygon at the time this method is called.

{

ofstream ofile(a\_fname, ios::app);

if ( !ofile.is\_open() )

{

cout << "Cannot open output file for rodenticide poly data " << a\_fname << endl;

char ch;

cin >> ch;

exit(1);

}

int noPoly=m\_land->SupplyLargestPolyNumUsed()+1;

double\* polyR = new double [noPoly];

for (int p=0; p<(noPoly); p++) polyR[p]=0.0;

int xext = m\_land->SupplySimAreaWidth();

int yext = m\_land->SupplySimAreaHeight();

for (int y=0; y<yext; y++)

{

for (int x=0; x<xext; x++)

{

double Rdt = m\_land->SupplyRodenticide(x,y);

int pindex = m\_land->SupplyPolyRefIndex(x,y);

polyR[pindex]+=Rdt;

}

}

ofile << endl; // Got to be here to easily allow an eof test later

for (int p=0; p<(noPoly-1); p++)

{

ofile << polyR[p] << '\t';

}

ofile << polyR[noPoly-1];

ofile.close();

delete polyR;

}

|  |  |  |  |
| --- | --- | --- | --- |
| void RodenticideManager::SetMap | ( | int | *a\_x*, |
|  |  | int | *a\_y*, |
|  |  | double | *a\_p* |
|  | ) |  |  |

inline

Set poisoned mice at x,y.

{ m\_pmouse\_map\_main[a\_y\*m\_width + a\_x] = a\_p; }

|  |  |  |  |
| --- | --- | --- | --- |
| void RodenticideManager::SetMapTwin | ( | int | *a\_x*, |
|  |  | int | *a\_y*, |
|  |  | double | *a\_p* |
|  | ) |  |  |

inline

Set poisoned mice at x,y on twin map.

{ m\_pmouse\_map\_twin[a\_y\*m\_width + a\_x] = a\_p; }

|  |  |  |  |
| --- | --- | --- | --- |
| void RodenticideManager::SetMapTwinMult | ( | int | *a\_x*, |
|  |  | int | *a\_y*, |
|  |  | double | *a\_p* |
|  | ) |  |  |

inline

Multiply poisoned mice at x,y on twin map by constant.

{ m\_pmouse\_map\_twin[a\_y\*m\_width + a\_x] \*= a\_p; }

|  |  |  |  |
| --- | --- | --- | --- |
| void RodenticideManager::SetMapTwinP | ( | int | *a\_x*, |
|  |  | int | *a\_y*, |
|  |  | double | *a\_p* |
|  | ) |  |  |

inline

Add to poisoned mice at x,y on twin map.

{ m\_pmouse\_map\_twin[a\_y\*m\_width + a\_x] += a\_p; }

|  |  |  |  |  |  |
| --- | --- | --- | --- | --- | --- |
| bool RodenticideManager::ShouldPlaceBait | ( | TTypesBaitLocation | *a\_BLt* | ) |  |

Place bait dependent upon a frequency test.

References cfg\_rodenticide\_BLcountry\_AnnFreq, cfg\_rodenticide\_BLtown\_AnnFreq, cfg\_rodenticide\_BLwoodland\_AnnFreq, g\_rand\_uni, tbl\_country, tbl\_town, tbl\_woodland, and CfgFloat::value().

{

#ifdef \_\_RODENTICIDE\_DISTRIBUTIONTEST

return true;

#endif

double chance = g\_rand\_uni();

switch (a\_BLt)

{

case tbl\_town:

if (chance < cfg\_rodenticide\_BLtown\_AnnFreq.value()) return true;

break;

case tbl\_country:

if (chance < cfg\_rodenticide\_BLcountry\_AnnFreq.value()) return true;

break;

case tbl\_woodland:

if (chance < cfg\_rodenticide\_BLwoodland\_AnnFreq.value()) return true;

break;

}

return false;

}

|  |  |  |  |  |  |
| --- | --- | --- | --- | --- | --- |
| void RodenticideManager::Tick | ( | void |  | ) |  |

Advance one day.

Check for bait location placement each day, but also do intialisation on Jan 1st.

For each bait location - first add immigration to the main map.

Then calculate diffusion for every cell - this is time consuming! Diffusion is added to twin map.

Apply mortalities to all cells in the twin map.

Finally swap the twin map and main map, then clear the twin map.

References cfg\_rodenticide\_dumpmapday, cfg\_rodenticide\_dumpmapstartyear, cfg\_rodenticide\_dumppolyendyear, cfg\_rodenticide\_dumppolyinterval, cfg\_rodenticide\_dumppolystartyear, and CfgInt::value().

Referenced by Landscape::Tick().

{

DoPlaceBait();

DoImmigration();

DoDiffuse();

DoDeath();

m\_pmouse\_map\_twin.swap(m\_pmouse\_map\_main); // Swap the twim map to the main map

m\_pmouse\_map\_twin.assign((m\_width+1) \* (m\_height+1), 0); // clears the twin map

// OUTPUT

int year = m\_land->SupplyYearNumber();

int today = m\_land->SupplyDayInYear();

if ((cfg\_rodenticide\_dumpmapstartyear.value()>=year) && (cfg\_rodenticide\_dumpmapstartyear.value()<=year) &&(cfg\_rodenticide\_dumpmapday.value()==today) )

{

DumpRodenticideMap("RodenticideMap.txt");

}

if ((year >= cfg\_rodenticide\_dumppolystartyear.value()) && (year <= cfg\_rodenticide\_dumppolyendyear.value()) )

{

if (today % cfg\_rodenticide\_dumppolyinterval.value()==0) RecordRodenticidePolyData("RodenticidePolyData.txt");

}

}

---

## Member Data Documentation

vector<BaitLocation> RodenticideManager::m\_BaitList

protected

List of bait locations.

double RodenticideManager::m\_deathRate

protected

poisoned mice death rate

double RodenticideManager::m\_diffusionRate

protected

poisoned mice diffusion rate

int RodenticideManager::m\_gridsize

protected

rodenticide grid size - also affect diffusion rate

int RodenticideManager::m\_height

protected

int RodenticideManager::m\_heightm1

protected

double RodenticideManager::m\_immigrationRate

protected

poisoned mice immigration rate

Landscape\* RodenticideManager::m\_land

protected

Landscape pointer.

vector<double> RodenticideManager::m\_pmouse\_map\_main

protected

Map of probability of encountering a poisoned mouse.

vector<double> RodenticideManager::m\_pmouse\_map\_twin

protected

Temporary map for calculation poisoned mouse diffusion.

int RodenticideManager::m\_seasonstartprob[tbl\_foobar][1000]

protected

season start probability distribution

int RodenticideManager::m\_width

protected

Landscape dimensions.

---

The documentation for this class was generated from the following files:

- Rodenticide.h
- Rodenticide.cpp


- RodenticideManager
- Generated on Thu Jan 10 2013 13:15:37 for ALMaSS Skylark ODdox by
   1.8.1.1
